# Unusual Cause of Sublingual Hematoma Compromising Upper Airway: Acquired Hemophilia A

**DOI:** 10.7759/cureus.113492

**Published:** 2026-07-28

**Authors:** Saho Miyake, Yuji Okazaki, Toshihisa Ichiba

**Affiliations:** 1 Emergency Medicine, Osaka Red Cross Hospital, Osaka, JPN; 2 Emergency Medicine, Hiroshima City Hiroshima Citizens Hospital, Hiroshima, JPN

**Keywords:** acquired hemophilia a, bypassing agent, difficult airway management, sublingual hematoma, upper airway obstruction

## Abstract

Sublingual hematoma is an uncommon but potentially life-threatening cause of upper airway obstruction. While most cases result from trauma, surgery, or anticoagulant use, acquired hemophilia A (AHA) is a rare autoimmune bleeding disorder caused by factor VIII inhibitors that may, in rare cases, present with spontaneous sublingual mucosal bleeding. We report a rare case of sublingual hematoma due to AHA that narrowed the upper airway.

A 90-year-old woman without a history of spontaneous bleeding presented to the emergency department (ED) with acute sublingual swelling and dysphagia. She had no history of trauma, recent dental procedures, or anticoagulant use. Physical examination revealed hoarseness and purplish swelling of the sublingual and submandibular regions. Laboratory examinations showed isolated prolongation of activated partial thromboplastin time (aPTT). Contrast-enhanced computed tomography revealed a sublingual hematoma with airway narrowing. Given the risk of airway obstruction, we prepared for awake nasotracheal intubation. A mixing study demonstrated failure of aPTT correction, and factor VIII activity was undetectable, strongly suggesting AHA. Because the hematoma enlarged during the first 12 hours after ED admission, we initiated intravenous administration of activated prothrombin complex concentrate. The hematoma subsequently improved. A positive factor VIII inhibitor confirmed the diagnosis of AHA, and immunosuppressive therapy with glucocorticoids was started. The aPTT normalized within two weeks, and the inhibitor became undetectable after three weeks. She was discharged on day 41 without additional bleeding events.

AHA should be considered in cases of spontaneous sublingual hematoma, particularly when accompanied by isolated aPTT prolongation. In such cases, because the hematoma may rapidly progress to cause upper airway obstruction, clinicians should initiate hemostatic therapy promptly when AHA is strongly suspected, even before a definitive diagnosis is established.

## Introduction

Sublingual hematoma is an uncommon but potentially life-threatening cause of upper airway obstruction that may present abruptly in the emergency department (ED) [1,2]. To address upper airway obstruction, clinicians need to rapidly identify the underlying cause of this condition and secure the airway. Most cases of sublingual hematoma are caused by trauma [3], postoperative bleeding [4], or excessive anticoagulation therapy [1,2,5]. Accordingly, the etiology is usually evident from the medical history or laboratory findings [1-5].

Acquired hemophilia A (AHA) is a rare autoimmune bleeding disorder caused by the development of autoantibodies against factor VIII [6]. It usually manifests as spontaneous soft-tissue or mucosal bleeding in elderly patients without a prior history of bleeding [6]. Although most bleeding episodes are not directly fatal, acute hemorrhage in the upper airway, including sublingual hematoma, is a rare but possibly life-threatening condition if not promptly recognized [7,8]. However, because sublingual hematoma due to AHA is uncommon, the challenges of recognition and diagnosis in an emergency setting and the optimal timing of treatment remain poorly understood.

We present a case of acute-onset sublingual hematoma due to AHA that led to upper airway compromise. This case highlights the importance of considering AHA as a potential cause of spontaneous sublingual hematoma in an emergency setting because timely diagnosis and appropriate hemostatic therapy can be life-saving.

## Case presentation

**Figure 1:**
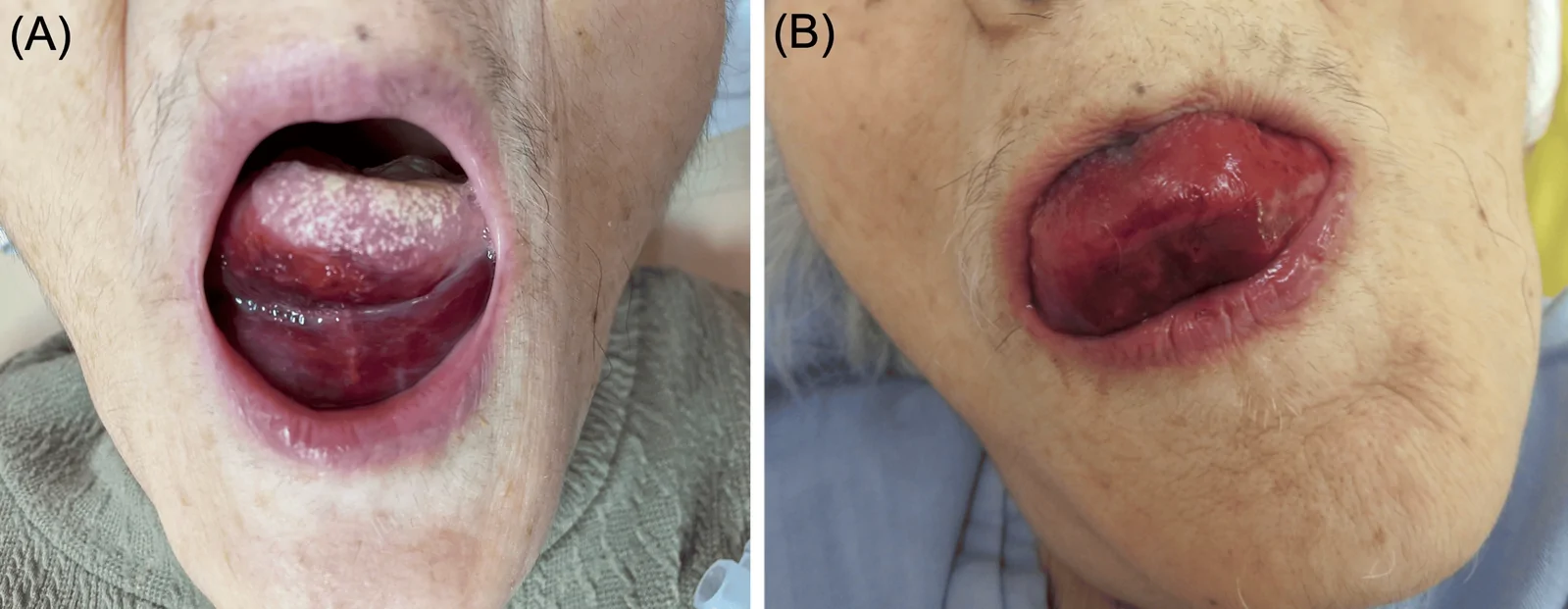
Oral examination showing sublingual hematoma (A) Swelling of the floor of the mouth with a purplish discoloration indicated a sublingual hematoma. (B) Twelve hours after admission, the sublingual hematoma had further progressed.

A 90-year-old woman presented with swelling of the sublingual space and dysphagia that had developed 10 hours prior to ED admission. She had a history of hypertension, hypothyroidism, and advanced dementia, and resided in a nursing home. She had no history of oral trauma, dental procedure, or use of anticoagulant agents. On arrival, her vital signs were: body temperature, 37.4 ℃; blood pressure, 104/60 mmHg; heart rate, 89 beats per minute; and oxygen saturation, 97 % without oxygen supplementation. Physical examination revealed swelling of the floor of the mouth with a purplish discoloration and painless swelling of the lower jaw (Figure 1A), which progressively enlarged over the following hours (Figure 1B).

No strider was noted, but hoarseness was present. Laboratory examinations showed mild anemia, a normal platelet count, normal prothrombin time-international normalized ratio (PT-INR) and D-dimer levels, and isolated prolongation of activated partial thromboplastin time (aPTT) (Table 1).

**Table 1:** Laboratory examinations AST: aspartate transferase; ALT: alanine transaminase; LD: lactate dehydrogenase; PT-INR: prothrombin time-international normalized ratio; aPTT: activated partial thromboplastin time.

	Hospital admission	Reference range
White blood counts	6800	3.3 - 8.6 x 10^3^/μL
Hemoglobin	7.3	13.7 - 16.8 g/dL
Platelet	30.5	15.8 - 34.8 x 10^4^/μL
AST	17	13 - 30 U/L
ALT	6	10 - 42 U/L
LD	212	124 - 222 U/L
Blood urea nitrogen	27	8 - 20 mg/dL
Creatinine	1.27	0.65 - 1.07 mg/dL
Creatinine kinase	27	59 - 248 U/L
C-reactive protein	0.711	< 0.14 mg/dL
Sodium	137.1	138 - 145 mmol/L
Potassium	5.1	3.6 - 4.8 mmol/L
Chloride	109.1	101 - 108 mmol/L
PT-INR	1.19	
aPTT	153.1	24 - 34 seconds
D-dimer	< 0.5	< 0.5 μg/mL

**Figure 2:**
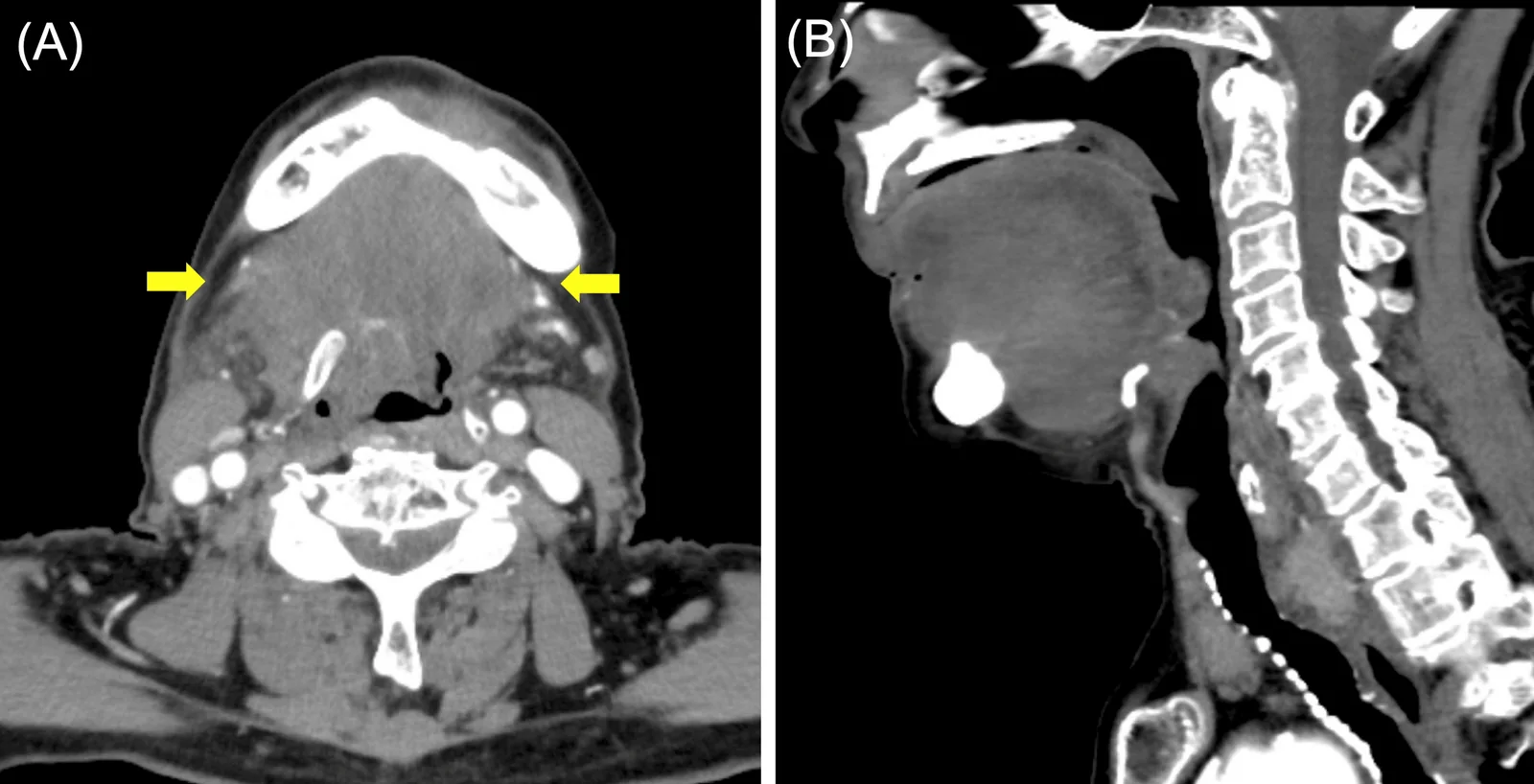
Contrast-enhanced computed tomography (A) Axial contrast-enhanced computed tomography (CT) shows soft tissue swelling extending from the tongue to the floor of the mouth, suggesting a sublingual hematoma (yellow arrow). (B) Coronal contrast-enhanced CT shows the narrowed upper airway.

Contrast-enhanced computed tomography (CT) showed soft tissue swelling extending from the tongue to the floor of the mouth, suggesting a sublingual hematoma, without evidence of any tumorous, pseudoaneurysm, or contrast extravasation (Figure 2).

Although we suspected acquired bleeding disorders, a mixing study and other coagulation factor assays (e.g., factor VIII activity) could not be performed immediately because it was nighttime. Based on her status and images, we considered the possibility of upper airway obstruction and prepared equipment for awake nasotracheal intubation and surgical airway protection in the intensive care unit.

Twelve hours after admission, the sublingual hematoma had further enlarged (Figure 1B). The mixing study performed 18 hours after admission revealed failure to acquire aPTT correction, suggesting the presence of coagulation factor inhibitors. Factor VIII activity was below the limit of detection, while von Willebrand factor and factor IX activities were normal. Lupus anticoagulant was within normal limits. We suspected the possibility of AHA, and intravenously administered an activated prothrombin complex concentrate [factor eight inhibitor bypassing activity (FEIBA); 80 U/kg/day)] 21 hours after admission. Subsequently, the sublingual hematoma gradually improved. A factor VIII inhibitor titer of 6 Bethesda units finally confirmed the diagnosis of AHA. Immunosuppressive therapy with prednisolone 40 mg per day was initiated on hospital day one. The aPTT normalized after two weeks, and the inhibitor became undetectable after three weeks. She was discharged on day 41 without additional bleeding events. At discharge, the prednisolone dose was 17.5 mg per day. Three months after discharge, there had been no recurrence of bleeding, and remission was being maintained with a prednisolone dose of 15 mg per day.

## Discussion

AHA may occasionally present with a sublingual hematoma that leads to upper airway obstruction. The most common bleeding sites in AHA are subcutaneous tissues and muscles [9]. Bleeding that is limited to these sites is generally not fatal; however, in fewer than 10% of cases, life-threatening hemorrhages can occur, typically involving the intracranial, pulmonary, gastrointestinal, or retroperitoneal regions [9]. Although oral cavity bleeding accounts for less than 1% of all AHA-related hemorrhages, sublingual hematoma represents a clinically critical condition because it can rapidly compromise the airway. While a few cases of sublingual hematoma due to AHA have been reported, AHA may remain an underrecognized cause of spontaneous sublingual hematoma overall [7,8]. Considering that AHA is a treatable condition, clinicians should always include it in the differential diagnosis of spontaneous sublingual hematoma. In our case, the key clue that led to the early suspicion of AHA was the occurrence of bleeding at an unusual site in combination with an isolated prolongation of aPTT. In patients with a bleeding tendency who exhibit a normal PT but a prolonged aPTT, without a history of congenital bleeding disorders or exposure to anticoagulants, a mixing study and evaluation of factor VIII activity should be promptly performed to identify acquired coagulation factor inhibitors [6]. Early recognition based on these findings enables timely diagnosis and may prevent subsequent life-threatening hemorrhage.

Upper airway compromise caused by AHA-induced sublingual hematoma may require special consideration for airway management. Two major points differ from standard difficult airway management. First, in cases of sublingual hematoma, the hematoma may extend to the base of the tongue, as in our case, making laryngoscopic visualization challenging. In such situations where the oral route is limited, awake nasotracheal intubation is often preferred even in emergency settings [10]. Although the usefulness of awake nasotracheal intubation in similar situations has been reported, there is insufficient evidence regarding its safety and efficacy [11]. In addition, patients with untreated AHA may have a high risk of epistaxis, which is a common complication of nasotracheal intubation [10]. Therefore, if this approach is attempted, it should be performed only by an experienced operator with adequate preparation and equipment. Second, front-of-neck access to secure the airway in patients with untreated AHA may carry a risk of uncontrollable bleeding and unexpected complications. In cases of diagnosed AHA, the preemptive administration of bypassing agents may help reduce bleeding risk during the surgical procedure [6]. However, in cases where AHA has not yet been diagnosed, the optimal timing and use of bypassing agents before surgical intervention is unclear. Therefore, the accumulation of additional reports of cases with undiagnosed but suspected AHA, such as ours, is needed to guide future decision-making regarding airway management strategies and the timing of hemostatic therapy.

## Conclusions

Clinicians should consider the possibility of AHA in patients with sublingual hematoma of unclear etiology. If bleeding remains uncontrolled, progressive hematoma expansion may lead to airway obstruction. Therefore, even before the diagnosis is confirmed, clinicians should prioritize timely and safe airway management and initiate hemostatic therapy with appropriate bypassing agents after obtaining diagnostic testing for AHA as early as possible.
